# Occupational Factors and Labor Market Outcomes Among Individuals with Sickness Absence due to Common Mental Disorders: A Population-Wide Cohort Study

**DOI:** 10.1007/s10926-025-10348-6

**Published:** 2025-11-27

**Authors:** Gerda Stutaite, Ellenor Mittendorfer-Rutz, Magnus Helgesson, Kristin Farrants, Katalin Gémes

**Affiliations:** 1 Division of Insurance Medicine, Department of Clinical Neuroscience, Karolinska Institutet, 171 77 Stockholm, Sweden; 2 Department of Public Health and Caring Sciences, Uppsala University, Box 564, 751 22 Uppsala, Sweden

**Keywords:** Depression, Anxiety disorders, Stress disorders, Sick leave, Disability pension, Job strain

## Abstract

**Purpose:**

To investigate how job demands, control, strain, and occupational sector and branch affect labor market outcomes following sickness absence (SA) due to common mental disorders (CMDs).

**Methods:**

This nationwide register-based cohort study included all residents in Sweden aged 25–55 who began a new > 30-day SA spell due to a CMD (ICD-10: F32-33, F40-43) in 2011–2013 (*n* = 79,673). Occupational sector and branch were identified through registers, and job demands, control, and strain were assessed using the Swedish Job Exposure Matrix. We used multinomial logistic regression to estimate associations between occupational factors and different unemployment and SA/disability pension (DP) durations during a three-year follow-up.

**Results:**

Public sector workers were less likely to have > 180 unemployment days (OR = 0.3, 95% CI 0.31–0.35). Working in education and public administration and in health and social services was associated with a lower likelihood of > 180 unemployment days, but a higher likelihood of > 365 SA/DP days. Low-control, passive (low control/low demands), and high-strain (low control/high demand) jobs were associated with an increased likelihood of both > 180 unemployment days and > 365 SA/DP days. For > 180 unemployment days, the ORs were 1.7 (95% CI 1.62–1.82) for low-control, 1.8 (95% CI 1.70–1.98) for passive, and 1.4 (95% CI 1.23–1.54) for high-strain jobs. For > 365 SA/DP days, the ORs were 1.3 (95% CI 1.22–1.34), 1.3 (95% CI 1.22–1.41), and 1.3 (95% CI 1.15–1.39), respectively.

**Conclusion:**

Particularly among individuals with SA due to CMDs, job demands, control, and strain are associated with future labor market exclusion and may be important targets for intervention.

**Supplementary Information:**

The online version contains supplementary material available at https://doi.org/10.1007/s10926-025-10348-6.

## Introduction

Diagnoses of common mental disorders (CMDs), including depression, anxiety, and stress-related disorders, have increased significantly in recent decades in Sweden and other OECD countries, constituting a major public health challenge [1, 2]. It is estimated that around 20% of the working-age population has a clinically diagnosed mental disorder, the majority of which are CMDs [1]. Due to their negative effect on work ability [3], CMDs are also the leading cause of sickness absence in Sweden and other OECD countries [1, 4]. For instance, in Sweden, CMDs accounted for approximately 90% of all psychiatric sickness absence spells exceeding 14 days beginning in 2018 and 2019 [5]. Most sickness absence spells due to CMDs last around 40 days; however, individuals with certain stress-related disorders may have sickness absence lasting for half a year or longer [6]. Consequently, CMDs not only cause suffering to the affected individuals but also pose a substantial economic burden for society [1].

The primary purpose of a sickness absence period is to allow individuals with CMDs, whose work ability has been compromised, to temporarily take time off work to seek timely care and prevent the progression of the disorder by providing temporary financial support [7]. However, sickness absence, particularly when prolonged, can contribute to long-term labor market exclusion [8–10] and worsening health [11]. Employment, in contrast, promotes long-term financial stability, social connectedness, a sense of identity, and better overall health [12]. Notably, studies have shown that many individuals with CMDs can remain in employment, despite their condition [13, 14]. Therefore, it is important to understand the factors that influence not only the risk of sickness absence among individuals with CMDs but also affect their labor market outcomes following a sickness absence spell due to CMDs. Occupational factors, particularly those related to the psychosocial work environment, are often modifiable and can be targeted in interventions to improve the health of individuals with CMDs, facilitating their return to work, and consequently reducing societal costs.

In occupational stress research, the psychosocial work environment is often evaluated using the job strain model, developed by Karasek and Theorell [15, 16]. The model focuses on the interaction between job demands and job control, defined as job strain, and suggests that high job demands (e.g., high workload and time pressures) combined with low job control (e.g., limited decision authority) may lead to mental distress. Working in high-strain jobs (high job demands and low job control) is associated with an increased risk of a CMD [17] and higher rates of sickness absence due to CMDs [18–21]. In contrast, some studies have shown that jobs characterized by low demands and low control are associated with higher rates of sickness absence in the general population [22], while a recent study suggested that the relationship between job strain and sickness absence may vary depending on the specific characteristics of an occupational group [23].

Knowledge about how job strain influences future labor market outcomes following a sickness absence episode due to CMDs remains especially scarce. A 2018 scoping review reported insufficient evidence—defined as fewer than three qualifying studies—regarding the impact of job demands, job control, and job strain on labor market outcomes, such as recurrent sickness absence and return to work among individuals on sickness absence due to CMDs [18]. Nevertheless, some studies have shown that higher psychosocial hazards and increased psychological and emotional demands at work are associated with delayed return to work in this population [24, 25]. However, these studies are relatively small and primarily examine time to return to work, rather than a broader range of labor market outcomes following a sickness absence spell due to a CMD. Thus, the impact of job demands, control, and strain on future labor market outcomes in this particular group is not yet fully understood.

Regarding the role of structural occupational factors, such as sector and branch, research has shown that public sector workers have a higher risk of CMDs than private sector workers, with the risk being especially high among individuals working in health and social services [26]. Similarly, public sector workers were also found to be at a higher risk of sickness absence and disability pension due to CMDs than private sector workers [27–29]. However, little is known about how working in the public versus private sector and across different occupational branches affects labor market outcomes following a period of sickness absence due to CMDs [13].

Therefore, this study aims to investigate how differences in job demands, control, and strain, and occupational sector and branch affect labor market outcomes among individuals with a sickness absence spell due to a CMD. To gain a more comprehensive understanding of how these individuals might be excluded from the labor market, we draw on the social insurance perspective, which conceptualizes labor market marginalization as periods of both unemployment and work disability, the latter primarily driven by illness or injury and measured through sickness absence and disability pension.

This study is the first population-based study of its scale using high-quality register data that includes detailed sociodemographic, socioeconomic, and health-related factors to assess the impact of occupational factors on a range of labor market outcomes following a sickness absence spell due to CMDs. The findings of this study could contribute valuable knowledge needed to inform future policy initiatives aimed at facilitating sustainable labor market participation for these individuals.

## Methods

### Study Data, Design, and Population

We conducted a register-based prospective cohort study, including all Swedish residents, aged 25–55, who were in gainful employment and started a new sickness absence (SA) spell due to a CMD lasting longer than 30 days between 2011 and 2013, and who had lived in Sweden for at least two years before the start of the study. CMD diagnoses were defined according to the International Classification of Diseases (ICD-10) codes: F32-F33 for depressive disorders, F40-41 for anxiety disorders, F42 for obsessive–compulsive disorder (OCD), and F43 for stress-induced disorders. Gainful employment status was defined by Statistics Sweden [30]. The earliest spell was designated the index spell for individuals with multiple SA spells matching the inclusion criteria.

Due to the low likelihood of return to work and high risk of permanent disability, we excluded individuals with a severe psychiatric or neurological disorder diagnosed in specialized outpatient or inpatient care (ICD-10) in the two years before the start of the study, or anytime during the study’s follow-up (Online Resource). These conditions were psychosis spectrum disorders (F20-29), bipolar disorder (F30-F31), early-onset neurodegenerative disorders (F00-F03 or G30-G32), anorexia nervosa (F50.0), and intellectual disabilities (F70-F79). We further excluded individuals with SA or disability pension (DP) due to any condition one year before the start of the index SA spell and individuals with missing information on their occupation, occupational sector, or branch (*n* total excluded = 37,967).

### Data Sources

We used the following national Swedish registers and databases, which are linked together by a unique identification number assigned to each individual: the “Micro-Data for Analyses of Social Insurance (MiDAS)” [31], the “Longitudinal Integration Database for Health Insurance and Labor Market Studies (LISA)” [30], the National Patient Register [32], the Causes of Death Register [33], and the Prescribed Drug Register [34]. A description of information used from each of the data sources is provided in the Online Resource.

### Sweden’s Social Security

Swedish residents with qualifying income from work or benefits related to parental leave or unemployment can receive SA compensation if their work capacity is impaired due to illness or injury [4]. For employed individuals, during the initial 14 days of sickness, the employer pays the SA benefits, reaching up to 80% of lost qualifying earnings [4]. The Swedish Social Insurance Agency disburses subsequent benefits [4]. For the unemployed, the Swedish Social Insurance Agency pays benefits from the second SA day [4]. Consequently, as information for this study is collected through the Swedish Social Insurance Agency, only SA spells lasting longer than 14 days were included.

Additionally, Swedish residents aged 19 to 66 with an illness or disability that impairs their ability to work for an extended period may qualify for DP benefits [4]. Individuals aged 19–29 may receive temporary activity compensation either due to decreased work ability or because they could not complete compulsory education in time, while those aged 30–66 may receive permanent compensation [4].

Individuals can receive either full (100%) or partial (75%, 50%, or 25%) SA and DP benefits [4].

In addition to public SA and DP benefits, individuals in Sweden may be entitled to supplementary sickness insurance based on collective agreements [35]. This collectively bargained insurance is negotiated between employer organizations and labor unions and varies across sectors and occupations: central government, municipal and regional, private white-collar, and private blue-collar employees [35]. Its primary aim is to provide additional financial support to individuals whose work capacity is reduced due to illness or injury [35]. In Sweden, 100% of public sector employees are covered by collective agreements, and approximately 85% of private sector employees, with coverage remaining stable over the past two decades [36].

Unemployed Swedish residents above age 20, registered as job seekers with the Swedish Public Employment Service and capable of working, can receive basic unemployment benefits, while some may also qualify for additional income-based benefits [37].

Finally, in Sweden, employment protection is regulated by the Employment Protection Act, under which the termination of employment due to illness or injury is prohibited [38]. Additionally, employers have a general responsibility to support rehabilitation and return-to-work planning for affected employees [39]. Employment may be terminated in cases of permanent work disability, for which DP benefits are paid by the Swedish Social Insurance Agency [39].

### Exposures

The exposure variables in this study were job demands, control, and strain, as well as occupational sector and branch.

We assessed job demands, control, and strain using the Swedish Job Exposure Matrix (SweJEM) for the years 1997–2013 [40, 41]. It consists of occupational-level scores for psychosocial job demands and control derived from the Swedish Work Environment and Health surveys [40, 41]. In these surveys, job demands were measured using questions focused on workload, stress, and concentration required at work, while job control was assessed using questions focused on decision authority and work task repetitiveness [40, 41]. Individual self-reported values were averaged and assigned to all persons in a specific occupation, based on the Swedish version of the International Standard Classification of Occupations (ISCO-88), separately for men and women [40, 41].

We categorized job demands and control into low, medium, and high based on their tertile distributions [42]. Additionally, we derived job strain based on the interaction between job demands and control. It included (1) Low-strain (low demands/high control), (2) Passive (low demands/low control), (3) Active (high demands/high control), (4) High-strain (high demands/low control), and (5) Intermediate-strain jobs, which included the remaining job demands and control combinations (Table 1) [15]. We then assigned the SweJEM values for job demands, control, and strain to individuals in the study population based on their occupational code at the end of the year preceding the start of their index SA spell due to a CMD.

**Table 1:** Population baseline characteristics (*n* = 79,673)

Characteristic	*n*	%
Sex		
Female	56,906	71.4
Male	22,767	28.6
Age		
25–29	9481	11.9
30–34	12,511	15.7
35–39	15,384	19.3
40–44	15,319	19.2
45–49	13,936	17.5
50–55	13,042	16.4
Country of birth		
Sweden	69,125	86.8
Other Nordic	1774	2.2
Other European	4101	5.1
Rest of the World	4673	5.9
Education level		
Elementary (< 10 years)	6690	8.4
High school (10–12 years)	36,586	45.9
University or college (> 12 years)	36,397	45.7
Family composition		
Living without a partner and without children	26,011	32.7
Living without a partner and with children	9086	11.4
Living with a partner and without children	9982	12.5
Living with a partner and with children	34,594	43.4
Type of living area		
City	31,541	39.6
Town or suburban area	33,491	42.0
Rural area	14,641	18.4
Occupational sector		
Private	45,983	57.7
Public	33,690	42.3
Occupational branch		
Production, industrial, and resource-based:	10,965	13.8
Agriculture, forestry, and fishing	*373*	*0.5*
Manufacturing and extraction	*7147*	*9.0*
Energy supply and environmental activities	*594*	*0.7*
Construction	*2851*	*3.6*
Trade, transport, and storage:	12,438	15.6
Trade	*9106*	*11.4*
Transport and storage	*3332*	*4.2*
Information, financial, and business services:	14,459	18.1
Information and communication	*3070*	*3.9*
Finance and insurance activities	*1620*	*2.0*
Real estate activities	*830*	*1.0*
Business services	*8939*	*11.2*
Hospitality and personal services:	3639	4.6
Hotel and restaurant operations	*1827*	*2.3*
Cultural and personal services	*1812*	*2.3*
Education and public administration:	17,952	22.5
Public administration and defense	*5590*	*7.0*
Training and education	*12,362*	*15.5*
Health and social services	20,220	25.4
Job demands (SweJEM)		
Low	12,059	15.2
Medium	37,478	47.0
High	30,136	37.8
Job control (SweJEM)		
Low	26,656	33.5
Medium	30,621	38.4
High	22,396	28.1
Job strain (SweJEM)		
Low-strain jobs (low demands/high control)	186	0.2
Active jobs (high demands/high control)	12,302	15.5
Intermediate strain	57,541	72.2
Passive jobs (low demands/low control)	6371	8.0
High-strain jobs (high demands/low control)	3273	4.1
Any unemployment days		
Yes	6000	7.5
No	73,673	92.5
SA history		
0 days (no SA history)	67,750	85.0
0 days < SA history ≤ 30 days	4878	6.1
30 days < SA history ≤ 90 days	4183	5.3
90 days < SA history ≤ 180 days	1781	2.2
180 days < SA history	1081	1.4
DP history		
Yes	369	0.5
No	79,304	99.5
Index SA diagnosis		
Depressive disorders (F32-F33)	29,837	37.4
Anxiety disorders and OCD (F40-F41 and F42)	9937	12.5
Stress-induced disorders (F43)	39,899	50.1
Index SA grade		
Full-time (100%)	71,870	90.2
Part-time (75%)	1082	1.4
Part-time (50%)	5763	7.2
Part-time (25%)	958	1.2
Net index SA days		
30 days < SA days ≤ 90 days	40,806	51.2
90 days < SA days ≤ 180 days	17,601	22.1
180 days < SA days	21,266	26.7
Mental comorbidities		
Other CMDs and burn-out (F32-F33, F40-F41, F42, F33, and Z730)	3723	4.7
Substance use disorders (F10-F19)	1257	1.6
Other affective disorders (F34, F38, and F39)	180	0.2
Personality disorders (F60-F69)	342	0.4
Attention-deficit hyperactivity disorder (ADHD) (F90.0)	278	0.4
Other behavioral emotional disorders (F50-F59, excl. F50.0, F90.1-F98)	819	1.0
Other psychological developmental disorders and autism-spectrum disorder (F80–F89, excl. F84.3–F84.9)	69	0.1
Other mental comorbidities (F04-F09, F44-F48, and F99)	289	0.4
Somatic comorbidities		
Cancer (C00-D48)	4075	5.1
Endocrine, nutritional, and metabolic disorders (E00-E90)	3234	4.1
Neurological disorders (G00-G99, excl. G30-G32)	2884	3.6
Circulatory system disorders (I00-I99)	3191	4.0
Respiratory disorders (J00-J99)	3700	4.6
Musculoskeletal disorders (M00-M99)	7514	9.4
Other chronic somatic disorders	23,330	29.3
Medications		
Antidepressants (ATC: N06A)		
No dispensations	61,560	77.3
One dispensation	9920	12.4
Two or more dispensations	8193	10.3
Other psychotropic medications		
No dispensations	63,189	79.3
One or more dispensations	16,484	20.7

a. *ADHD* attention deficit hyperactivity disorder, *ATC* anatomical therapeutic chemical, *CMDs* common mental disorders, *DP* disability pension, *OCD* obsessive compulsive disorder, *SA* sickness absence, *SweJEM* The Swedish Job Exposure Matrix

b. Measurement timepoints: age, education level, family composition, type of living area, occupation, and occupational sector and branch were assessed on December 31 of the year before the index SA began. UE days were measured for the entire year preceding the start of the index SA spell. SA/DP history was assessed in the two-year period prior to the exclusion window, which began one year before the index SA. Mental and somatic comorbidities were assessed within two years of the start of the index SA spell, and medication use was assessed six months before and three months after the index SA spell started

c. Missing information was classified as the lowest education level—“elementary education (< 10 years)” (*n* = 186 (0.23%))

d. Intermediate job strain: low demands/medium control, medium demands/low control, medium demands/medium control, medium demands/high control, high demands/medium control

e. All diagnostic codes refer to the 10th version of the International Classification of Diseases (ICD-10) codes

f. Other chronic somatic disorders: infectious and parasitic diseases (ICD-10: A00-B99), diseases of the blood and blood-forming organs (ICD-10: D50-D89), diseases of the eye and ear (ICD-10: H00-H95), diseases of the digestive system (ICD-10: K00-K93), diseases of the skin and subcutaneous tissue (ICD-10: L00-L99), and diseases of the genitourinary system (ICD-10: N00-N99)

g. Other psychotropic medications: anxiolytics (ATC: N05B), hypnotics and sedatives (ATC: N05C), psychostimulants, ADHD agents, and nootropics (ATC: N06B), psycholeptics and psychoanaleptics in combination (ATC: N06C)

The specific occupational groups within each occupational branch are listed in italics

The occupational sector was divided into (1) Private and (2) Public, and the occupational branch was grouped into (1) Production, industrial, and resource-based branch (agriculture, forestry, fishing, manufacturing, extraction, energy supply, environmental services, and construction), (2) Trade, transport, and storage, (3) Information, financial, and business services (information, communication, finance, insurance, and real estate activities and business services), (4) Hospitality and personal services (hotel and restaurant operations and cultural and personal services), (5) Education and public administration (public administration and defense and training and education), and 6) Health and social services. We assessed the occupational sector and branch at the end of the year preceding the start of the index SA spell due to a CMD.

### Outcomes and Follow-Up

The two outcomes of interest in this study were the sum of unemployment days and SA/DP days during the follow-up period. SA days used to define the study population, and all following SA and DP days refer to net days, which means, for example, that two half-days are counted as one net day. We categorized unemployment days into (1) No (zero) unemployment days, (2) 0 < unemployment days ≤ 180, and (3) > 180 unemployment days, to assess both medium-term (up to 180 days) and long-term (more than 180 days) unemployment. Net SA/DP days were categorized into (1) 30 < SA/DP days ≤ 90 Days, (2) 90 < SA/DP days ≤ 180, (3) 180 < SA/DP days ≤ 365, and (4) > 365 SA/DP days, based on the National Board of Health and Welfare’s recommendations for SA durations for different CMDs, which consider specific diagnoses, disorder severity, and work ability [43].

We assessed unemployment days per calendar year for three consecutive years, beginning the year after the start of the index SA spell (Online Resource). SA/DP days, on the other hand, were assessed for three consecutive years, beginning on the first day of the index SA spell. Individuals who emigrated or died during the follow-up period were excluded.

### Covariates

Covariates assessed on December 31 of the year before the start of the index SA spell were age, education level, type of living area, and family composition (Table 1). SA/DP history was assessed in the two-year period prior to the exclusion window, which began one year before the index SA. Psychotropic medication dispensations were assessed six months before and three months after the first day of the index SA spell.

Additionally, based on ICD-10 codes from inpatient and specialized outpatient care, we identified mental and somatic comorbidities in the two years before the start of the index SA, detailed in Table 1. Finally, we assessed the duration, grade (part-time versus full-time), and diagnosis of the index SA spell.

### Statistical Analyses

First, we computed descriptive characteristics for the entire study population (Table 1, Online Resource). Then, we conducted separate multinomial logistic regression analyses to examine the associations between occupational factors and varying numbers of unemployment and SA/DP days during the three-year follow-up. The reference group for the unemployment analysis was no (zero) unemployment days, and for the SA/DP analysis, it was 30 < days ≤ 90. Crude and adjusted analyses were conducted for each outcome, with Model 1 adjusted for sex and age, Model 2 additionally adjusted for education, country of birth, and type of living area, and Model 3 further adjusted for index SA diagnosis, SA/DP history, and mental and somatic comorbidities. Odds ratios (ORs) with 95% confidence intervals (CIs) were calculated for each association.

Individuals who emigrated or died during the follow-up period were excluded (unemployment: *n* = 773 (0.97%) and SA/DP: *n* = 706 (0.89%)). We computed descriptive statistics to compare baseline characteristics between the individuals included in the unemployment analysis and the excluded ones (Online Resource). Additionally, we excluded individuals with low job strain (low demands/high control) from both job strain analyses (unemployment: *n* = 183 (0.23%) and SA/DP: *n* = 184 (0.23%)) due to low observation counts. To assess whether this exclusion altered the results, we conducted a sensitivity analysis that included those with low job strain (Online Resource).

We performed data management using SAS 9.4 and conducted data analysis using R.

## Results

### Descriptive Characteristics

The final study population consisted of 79,673 individuals, with a mean age of 40.3 years (standard deviation (SD) = 8.2) (Table 1). Most were females (71.4%) and born in Sweden (86.8%). About half had completed high school (45.9%), and 45.7% had a university education. 43.4% of individuals lived with a partner and children, while 32.7% lived without either. Most resided in towns or suburban areas (42.0%), while 39.6% lived in cities.

Regarding occupational factors, most individuals worked in the private sector (57.7%), and the most common occupational branches were health and social services (25.4%) and education and public administration (22.5%). Medium-demand (47.0%), medium-control (38.4%), and intermediate-strain (72.2%) jobs were the most common, while 15.5% worked in active (high demands/high control) jobs. Moreover, 92.5% of individuals had no unemployment days in the year before the start of the index SA spell. During follow-up, most (76.9%) remained without any unemployment days, and among those with at least one, the average was 184.1 days (SD = 180.1).

Regarding health-related factors, 15.0% individuals had SA/DP days in the two years before the start of the index SA spell, while only 0.5% had DP days in the same timeframe. The most common comorbidity in the two years before the study began was another CMD (4.7%). The index SA spell was predominantly full-time (90.2%), with stress-related disorders (ICD-10: F43) as the most common diagnosis (50.1%). During follow-up, individuals had an average of 245.2 SA/DP days (SD = 254.6).

### Unemployment

**Fig. 1:**
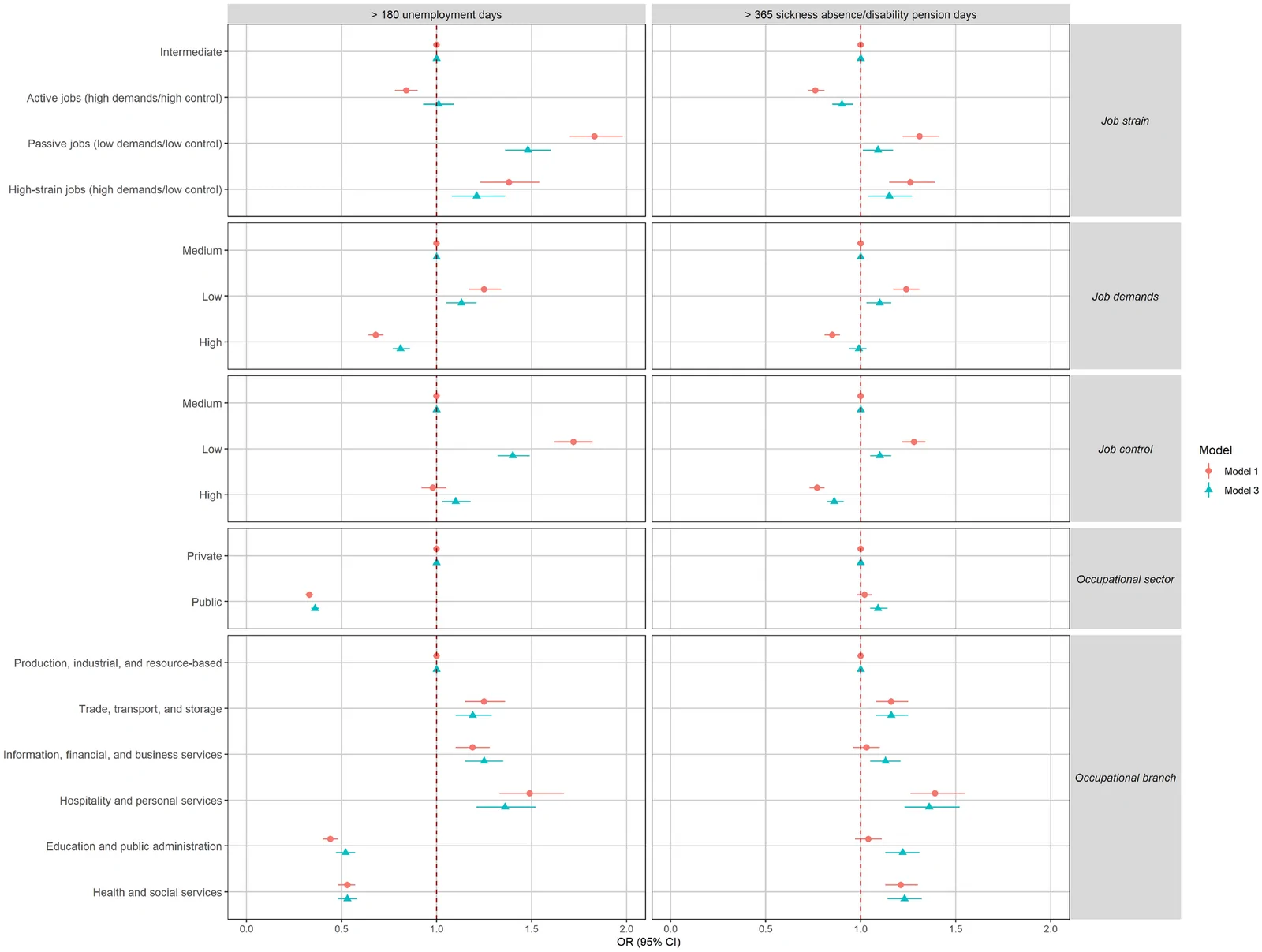
Odds ratio (OR) plots of the multinomial logistic regression models [*n* = 78,900 for unemployment and *n* = 78,967 for sickness absence and disability pension (SA/DP)]. Model 1 was adjusted for sex and age, and Model 3 was additionally adjusted for education, country of birth, type of living area, index SA diagnosis, SA/DP history, and mental and somatic comorbidities. Reference categories: 0 unemployment days for the unemployment analysis, and 30 < SA/DP days ≤ 90 for the SA/DP analysis

We found that, adjusted for age and sex, working in passive (low demands/low control) and high-strain (high demands/low control) jobs was associated with higher odds of having both up to 180 and > 180 days of unemployment compared to working in intermediate-strain jobs (Table 2, Fig. 1). For > 180 unemployment days, the OR was 1.8 (95% CI 1.70–1.98) for passive, and 1.4 (95% CI 1.23–1.54) for high-strain jobs. Results with further adjustments for socioeconomic and health-related factors are presented in Table 2. When job demands and job control were assessed separately, low job control was associated with higher odds of having > 180 unemployment days across all models compared to medium job control (OR = 1.7, 95% CI 1.62–1.82). In contrast, high job demands were inversely associated with both unemployment durations (OR = 0.7, 95% CI 0.64–0.72 for > 180 unemployment days).

**Table 2:** Multinomial logistic regression results for unemployment (UE) days (*n* = 78,900)

Occupational characteristic	Outcome category	Crude OR (95% CI)	Model 1 OR (95% CI)	Model 2 OR (95% CI)	Model 3 OR (95% CI)
Job strain					
Intermediate	0 < UE Days ≤ 180	1 (ref)	1 (ref)	1 (ref)	1 (ref)
	> 180 UE Days	1 (ref)	1 (ref)	1 (ref)	1 (ref)
Active jobs (high demands/high control)	0 < UE Days ≤ 180	0.79 (0.74–0.84)	0.79 (0.74–0.84)	0.88 (0.83–0.94)	0.91 (0.85–0.97)
	> 180 UE Days	0.89 (0.83–0.96)	0.84 (0.78–0.90)	0.98 (0.91–1.06)	1.01 (0.93–1.09)
Passive jobs (low demands/low control)	0 < UE Days ≤ 180	1.44 (1.34–1.54)	1.31 (1.22–1.41)	1.14 (1.06–1.23)	1.11 (1.03–1.20)
	> 180 UE Days	2.28 (2.11–2.45)	1.83 (1.70–1.98)	1.50 (1.39–1.62)	1.48 (1.36–1.60)
High-strain jobs (high demands/low control)	0 < UE Days ≤ 180	1.48 (1.34–1.62)	1.37 (1.25–1.51)	1.28 (1.17–1.41)	1.27 (1.15–1.39)
	> 180 UE Days	1.67 (1.50–1.87)	1.38 (1.23–1.54)	1.22 (1.09–1.37)	1.21 (1.08–1.36)
Job demands					
Low	0 < UE Days ≤ 180	1.17 (1.10–1.24)	1.07 (1.01–1.14)	1.01 (0.95–1.07)	0.98 (0.92–1.04)
	> 180 UE Days	1.55 (1.46–1.66)	1.25 (1.17–1.34)	1.15 (1.07–1.23)	1.13 (1.05–1.21)
Medium	0 < UE Days ≤ 180	1 (ref)	1 (ref)	1 (ref)	1 (ref)
	> 180 UE Days	1 (ref)	1 (ref)	1 (ref)	1 (ref)
High	0 < UE Days ≤ 180	0.72 (0.69–0.75)	0.73 (0.70–0.76)	0.83 (0.79–0.87)	0.84 (0.80–0.88)
	> 180 UE Days	0.69 (0.66–0.73)	0.68 (0.64–0.72)	0.80 (0.76–0.85)	0.81 (0.77–0.86)
Job control					
Low	0 < UE Days ≤ 180	1.41 (1.34–1.47)	1.36 (1.30–1.43)	1.20 (1.14–1.27)	1.18 (1.12–1.24)
	> 180 UE Days	1.79 (1.69–1.89)	1.72 (1.62–1.82)	1.42 (1.34–1.51)	1.40 (1.32–1.49)
Medium	0 < UE Days ≤ 180	1 (ref)	1 (ref)	1 (ref)	1 (ref)
	> 180 UE Days	1 (ref)	1 (ref)	1 (ref)	1 (ref)
High	0 < UE Days ≤ 180	0.85 (0.81–0.90)	0.83 (0.79–0.88)	0.89 (0.84–0.94)	0.91 (0.86–0.96)
	> 180 UE Days	1.06 (0.99–1.13)	0.98 (0.92–1.05)	1.08 (1.01–1.16)	1.10 (1.03–1.18)
Job sector					
Private	0 < UE Days ≤ 180	1 (ref)	1 (ref)	1 (ref)	1 (ref)
	> 180 UE Days	1 (ref)	1 (ref)	1 (ref)	1 (ref)
Public	0 < UE Days ≤ 180	0.44 (0.42–0.46)	0.45 (0.43–0.47)	0.47 (0.45–0.50)	0.47 (0.45–0.49)
	> 180 UE Days	0.30 (0.28–0.31)	0.33 (0.31–0.35)	0.36 (0.34–0.39)	0.36 (0.34–0.38)
Occupational branch					
Production, industrial, and resource-based	0 < UE Days ≤ 180	1 (ref)	1 (ref)	1 (ref)	1 (ref)
	> 180 UE Days	1 (ref)	1 (ref)	1 (ref)	1 (ref)
Trade, transport, and storage	0 < UE Days ≤ 180	1.28 (1.19–1.37)	1.27 (1.19–1.37)	1.26 (1.17–1.35)	1.26 (1.17–1.35)
	> 180 UE Days	1.17 (1.08–1.27)	1.25 (1.15–1.36)	1.19 (1.09–1.29)	1.19 (1.10–1.29)
Information, financial, and business services	0 < UE Days ≤ 180	1.10 (1.03–1.18)	1.11 (1.03–1.19)	1.18 (1.10–1.27)	1.18 (1.10–1.27)
	> 180 UE Days	1.08 (0.99–1.17)	1.19 (1.10–1.28)	1.25 (1.15–1.35)	1.25 (1.15–1.35)
Hospitality and personal services	0 < UE Days ≤ 180	1.47 (1.33–1.62)	1.46 (1.32–1.62)	1.44 (1.30–1.59)	1.42 (1.28–1.57)
	> 180 UE Days	1.33 (1.19–1.49)	1.49 (1.33–1.67)	1.37 (1.22–1.54)	1.36 (1.21–1.52)
Education and public administration	0 < UE Days ≤ 180	0.59 (0.55–0.63)	0.62 (0.57–0.67)	0.71 (0.65–0.76)	0.70 (0.65–0.76)
	> 180 UE Days	0.37 (0.34–0.40)	0.44 (0.40–0.48)	0.52 (0.47–0.57)	0.52 (0.47–0.57)
Health and social services	0 < UE Days ≤ 180	0.66 (0.61–0.71)	0.69 (0.64–0.74)	0.71 (0.66–0.77)	0.69 (0.64–0.75)
	> 180 UE Days	0.43 (0.40–0.47)	0.53 (0.48–0.57)	0.54 (0.49–0.59)	0.53 (0.48–0.58)

a. Model 1 was adjusted for sex and age, Model 2 was additionally adjusted for education, country of birth, and type of living area, and Model 3 was further adjusted for index SA diagnosis, SA/DP history, and mental and somatic comorbidities. Reference category: 0 UE days during the follow-up period

b. Individuals with low job strain (low demands/low control) were excluded due to low observation counts (*n* = 183 (0.23%))

c. Intermediate job strain: low demands/medium control, medium demands/low control, medium demands/medium control, medium demands/high control, high demands/medium control

d. Production, industrial, and resource-based branch: agriculture, forestry, fishing, manufacturing, extraction, energy supply, environmental services, and construction

e. Information and business services: information, communication, finance, insurance, and real estate activities and business services

### SA/DP

Similar to the unemployment analysis, working in passive and high-strain jobs was associated with an increased likelihood of having > 365 days of SA/DP (Table 3, Fig. 1). In the model adjusted for sex and age, the OR was 1.3 (95% CI 1.22–1.41) for passive and 1.3 (95% CI 1.15–1.39) for high-strain jobs. Active (high demands/high control) jobs, in contrast, showed an inverse association with the same outcome category (OR = 0.8, 95% CI 0.72–0.81 for > 365 days of SA/DP). Additionally, high job control, when assessed separately from job demands, was associated with lower odds of having > 365 SA/DP days compared to medium job control (OR = 0.8, 95% CI 0.73–0.81).

**Table 3:** Multinomial logistic regression results for sickness absence and disability pension (SA/DP) days (*n* = 78,967)

Occupational characteristic	Outcome category	Crude OR (95% CI)	Model 1 OR (95% CI)	Model 2 OR (95% CI)	Model 3 OR (95% CI)
Job strain					
Intermediate	90 < SA/DP Days ≤ 180	1 (ref)	1 (ref)	1 (ref)	1 (ref)
	180 < SA/DP Days ≤ 365	1 (ref)	1 (ref)	1 (ref)	1 (ref)
	> 365 SA/DP Days	1 (ref)	1 (ref)	1 (ref)	1 (ref)
Active jobs (high demands/high control)	90 < SA/DP Days ≤ 180	0.96 (0.91–1.01)	0.98 (0.93–1.03)	0.98 (0.93–1.04)	1.00 (0.95–1.06)
	180 < SA/DP Days ≤ 365	0.95 (0.90–1.00)	0.98 (0.93–1.03)	1.01 (0.95–1.06)	1.04 (0.98–1.10)
	> 365 SA/DP Days	0.75 (0.71–0.80)	0.76 (0.72–0.81)	0.85 (0.80–0.90)	0.90 (0.85–0.96)
Passive jobs (low demands/low control)	90 < SA/DP Days ≤ 180	0.92 (0.86–0.98)	0.98 (0.91–1.05)	0.98 (0.91–1.05)	0.96 (0.89–1.04)
	180 < SA/DP Days ≤ 365	0.88 (0.81–0.95)	1.00 (0.93–1.08)	0.98 (0.90–1.05)	0.95 (0.88–1.03)
	> 365 SA/DP Days	1.16 (1.08–1.24)	1.31 (1.22–1.41)	1.14 (1.06–1.23)	1.09 (1.01–1.17)
High-strain jobs (high demands/low control)	90 < SA/DP Days ≤ 180	0.99 (0.90–1.09)	1.05 (0.95–1.16)	1.05 (0.95–1.16)	1.04 (0.94–1.15)
	180 < SA/DP Days ≤ 365	0.94 (0.85–1.04)	1.05 (0.95–1.17)	1.04 (0.94–1.16)	1.02 (0.92–1.13)
	> 365 SA/DP Days	1.14 (1.03–1.25)	1.26 (1.15–1.39)	1.20 (1.08–1.32)	1.15 (1.04–1.27)
Job demands					
Low	90 < SA/DP Days ≤ 180	0.97 (0.91–1.02)	1.03 (0.97–1.09)	1.02 (0.97–1.08)	1.01 (0.95–1.07)
	180 < SA/DP Days ≤ 365	0.93 (0.87–0.98)	1.05 (0.99–1.12)	1.03 (0.97–1.09)	1.01 (0.95–1.07)
	> 365 SA/DP Days	1.10 (1.04–1.17)	1.24 (1.17–1.31)	1.15 (1.08–1.21)	1.10 (1.03–1.16)
Medium	90 < SA/DP Days ≤ 180	1 (ref)	1 (ref)	1 (ref)	1 (ref)
	180 < SA/DP Days ≤ 365	1 (ref)	1 (ref)	1 (ref)	1 (ref)
	> 365 SA/DP Days	1 (ref)	1 (ref)	1 (ref)	1 (ref)
High	90 < SA/DP Days ≤ 180	1.01 (0.97–1.05)	1.01 (0.97–1.06)	1.02 (0.98–1.07)	1.03 (0.99–1.08)
	180 < SA/DP Days ≤ 365	1.01 (0.96–1.05)	1.01 (0.97–1.05)	1.04 (0.99–1.09)	1.06 (1.01–1.11)
	> 365 SA/DP Days	0.85 (0.82–0.89)	0.85 (0.81–0.89)	0.95 (0.91–1.00)	0.99 (0.94–1.03)
Job control					
Low	90 < SA/DP Days ≤ 180	0.98 (0.94–1.03)	0.99 (0.95–1.04)	0.99 (0.94–1.04)	0.97 (0.93–1.02)
	180 < SA/DP Days ≤ 365	0.98 (0.93–1.02)	1.00 (0.96–1.05)	0.98 (0.93–1.03)	0.95 (0.91–1.00)
	> 365 SA/DP Days	1.23 (1.18–1.29)	1.28 (1.22–1.34)	1.15 (1.10–1.21)	1.10 (1.05–1.16)
Medium	90 < SA/DP Days ≤ 180	1 (ref)	1 (ref)	1 (ref)	1 (ref)
	180 < SA/DP Days ≤ 365	1 (ref)	1 (ref)	1 (ref)	1 (ref)
	> 365 SA/DP Days	1 (ref)	1 (ref)	1 (ref)	1 (ref)
High	90 < SA/DP Days ≤ 180	0.95 (0.90–0.99)	0.96 (0.92–1.01)	0.97 (0.92–1.01)	0.99 (0.94–1.03)
	180 < SA/DP Days ≤ 365	0.91 (0.87–0.96)	0.94 (0.90–0.99)	0.96 (0.92–1.01)	0.99 (0.94–1.04)
	> 365 SA/DP Days	0.75 (0.72–0.79)	0.77 (0.73–0.81)	0.81 (0.77–0.85)	0.86 (0.82–0.91)
Job sector					
Private	90 < SA/DP Days ≤ 180	1 (ref)	1 (ref)	1 (ref)	1 (ref)
	180 < SA/DP Days ≤ 365	1 (ref)	1 (ref)	1 (ref)	1 (ref)
	> 365 SA/DP Days	1 (ref)	1 (ref)	1 (ref)	1 (ref)
Public	90 < SA/DP Days ≤ 180	1.09 (1.05–1.13)	1.05 (1.00–1.09)	1.05 (1.01–1.09)	1.04 (1.00–1.09)
	180 < SA/DP Days ≤ 365	1.22 (1.18–1.27)	1.11 (1.07–1.16)	1.13 (1.09–1.18)	1.12 (1.07–1.17)
	> 365 SA/DP Days	1.12 (1.08–1.17)	1.02 (0.98–1.06)	1.11 (1.07–1.16)	1.09 (1.05–1.14)
Occupational branch					
Production, industrial, and resource-based	90 < SA/DP Days ≤ 180	1 (ref)	1 (ref)	1 (ref)	1 (ref)
	180 < SA/DP Days ≤ 365	1 (ref)	1 (ref)	1 (ref)	1 (ref)
	> 365 SA/DP Days	1 (ref)	1 (ref)	1 (ref)	1 (ref)
Trade, transport, and storage	90 < SA/DP Days ≤ 180	1.02 (0.95–1.09)	0.99 (0.92–1.06)	0.99 (0.93–1.06)	0.99 (0.93–1.06)
	180 < SA/DP Days ≤ 365	1.07 (0.99–1.15)	1.02 (0.95–1.10)	1.03 (0.95–1.10)	1.03 (0.95–1.11)
	> 365 SA/DP Days	1.19 (1.10–1.27)	1.16 (1.08–1.25)	1.16 (1.07–1.24)	1.16 (1.08–1.25)
Information, financial, and business services	90 < SA/DP Days ≤ 180	0.97 (0.91–1.03)	0.93 (0.88–1.00)	0.94 (0.88–1.01)	0.95 (0.89–1.01)
	180 < SA/DP Days ≤ 365	1.06 (0.98–1.13)	0.99 (0.92–1.06)	1.02 (0.95–1.10)	1.03 (0.96–1.10)
	> 365 SA/DP Days	1.07 (1.00–1.15)	1.03 (0.96–1.10)	1.13 (1.05–1.21)	1.13 (1.05–1.21)
Hospitality and personal services	90 < SA/DP Days ≤ 180	0.98 (0.88–1.08)	0.93 (0.84–1.03)	0.94 (0.85–1.04)	0.94 (0.85–1.04)
	180 < SA/DP Days ≤ 365	1.06 (0.95–1.18)	0.98 (0.88–1.10)	1.00 (0.89–1.11)	0.99 (0.89–1.10)
	> 365 SA/DP Days	1.45 (1.31–1.60)	1.39 (1.26–1.55)	1.40 (1.26–1.55)	1.36 (1.23–1.52)
Education and public administration	90 < SA/DP Days ≤ 180	1.10 (1.03–1.17)	1.02 (0.96–1.09)	1.04 (0.97–1.11)	1.04 (0.97–1.11)
	180 < SA/DP Days ≤ 365	1.24 (1.16–1.33)	1.08 (1.01–1.16)	1.13 (1.05–1.22)	1.13 (1.05–1.21)
	> 365 SA/DP Days	1.16 (1.08–1.24)	1.04 (0.97–1.11)	1.22 (1.14–1.32)	1.22 (1.13–1.31)
Health and social services	90 < SA/DP Days ≤ 180	1.08 (1.02–1.15)	1.00 (0.94–1.07)	1.01 (0.95–1.08)	0.99 (0.93–1.06)
	180 < SA/DP Days ≤ 365	1.29 (1.21–1.38)	1.11 (1.04–1.19)	1.14 (1.06–1.22)	1.10 (1.03–1.18)
	> 365 SA/DP Days	1.36 (1.28–1.46)	1.21 (1.13–1.30)	1.29 (1.20–1.38)	1.23 (1.14–1.32)

a. Model 1 was adjusted for sex and age, Model 2 was additionally adjusted for education, country of birth, and type of living area, and Model 3 was further adjusted for index SA diagnosis, SA/DP history, and mental and somatic comorbidities. Reference category: 30 < SA/DP Days ≤ 90 during the follow-up period

b. Individuals with low job strain (low demands/low control) were excluded due to low observation counts (*n* = 184 (0.24%))

c. Intermediate job strain: low demands/medium control, medium demands/low control, medium demands/medium control, medium demands/high control, high demands/medium control

d. Production, industrial, and resource-based branch: agriculture, forestry, fishing, manufacturing, extraction, energy supply, environmental services, and construction

e. Information, financial, and business services: information, communication, finance, insurance, and real estate activities and business services

### Occupational Sector and Branch

Adjusted for age and sex, public sector workers were less likely to have both up to 180 unemployment days and > 180 unemployment days (OR = 0.3, 95% CI 0.31–0.35 for > 180 unemployment days) compared with private sector workers (Table 2). This pattern was not observed for SA/DP days (Table 3). Additionally, working in education and public administration and in health and social services was associated with lower odds of having unemployment days. In the model adjusted for sociodemographic and health-related factors, for > 180 unemployment days, the OR was 0.5 (95% CI 0.47–0.57) for education and public administration, and 0.5 (95% CI 0.48–0.58) for health and social services. In contrast, individuals in these branches had higher odds of having > 365 SA/DP days during the follow-up (OR = 1.2, 95% CI 1.13–1.31 for education and public administration; OR = 1.2, 95% CI 1.14–1.32 for health and social services).

## Discussion

### Main Findings

This study examined how differences in job demands, control, strain, and occupational sector and branch affected labor market outcomes—unemployment and SA/DP—among individuals with SA due to CMDs. Our findings showed that in this population, high-strain (high demands/low control) and passive (low demands/low control) jobs were associated with both medium (up to 180 days) and long-term (> 180 days) unemployment and long-term (> 365 days) SA/DP. Working in active (high demands/high control) jobs, in contrast, was associated with a lower likelihood of having > 365 days of SA/DP. When job demands and job control were assessed separately, we found that individuals in low-control jobs were more likely to have medium and long-term unemployment and long-term SA/DP, while those in jobs with high demands were less likely to experience both medium and long-term unemployment.

Additionally, public sector workers were less likely to experience medium and long-term unemployment, but more likely to experience long-term SA/DP. Similarly, working in education and public administration, and in health and social services, was associated with a lower likelihood of medium and long-term unemployment, yet a higher likelihood of long-term SA/DP.

### Comparison with Literature

Direct comparisons with studies specifically analyzing labor market outcomes among individuals with SA due to CMDs are limited, as few such studies exist. However, we found that individuals in high-strain (high demands/low control) jobs were more likely to have long-term SA/DP following the initial SA spell, which aligns with previous findings suggesting that high psychosocial hazards—a concept comparable to high job strain, defined as high workload and emotional demands combined with low decision authority—are associated with longer SA durations among individuals with CMDs [24]. Our results are also consistent with previous studies showing that high-strain jobs are associated with an increased risk of SA and DP among individuals with CMDs [18–21, 44].

Additionally, we found that working in passive (low demands/low control) jobs was associated with a higher likelihood of having long-term SA/DP following SA due to CMDs, while working in active (high demands/high control) jobs was associated with a lower likelihood. These findings are consistent with some previous research showing lower SA risk among individuals with CMDs in active jobs [14]. According to Karasek’s job strain model, active jobs combine a challenging workload with the opportunity to exercise decision-making and autonomy, which can contribute to professional growth, increase job satisfaction, and reduce depression [15, 16]. Conversely, passive jobs have been associated with increased rates of depression [45]. However, the relationship between active and passive jobs and the risk of CMDs, SA, and SA due to CMDs remains not fully understood, with several studies reporting inconclusive results, particularly when comparing men and women [42, 46]. Specifically among women, some findings suggest that working in passive jobs is associated with an increased risk of SA/DP [42], while others show that working in active jobs may contribute to increased rates of long-term SA [46].

Moreover, several studies assessed job demands and job control independently of each other and found associations between high job demands and delayed return to work among individuals with SA due to CMDs [25, 47]. In our study, working in high-demand jobs was not associated with any of the examined SA/DP durations following the initial SA spell due to CMDs. However, we found that individuals in low-control jobs were more likely to experience long-term SA/DP compared to those in medium-control jobs. This suggests that low job control might be a stronger predictor of potentially detrimental labor market outcomes than high job demands when assessed separately. While the populations examined are not directly comparable, our findings are consistent with some previous studies suggesting a protective effect of increased job control against SA among individuals with CMDs [48] and against delayed return to work in studies involving individuals already on SA due to CMDs [47, 49]. Thus, future research should continue to examine both dimensions of job strain—job demands and control—to identify aspects that may promote return to work and reduce SA rates in this population.

It is worthwhile noting that in this study, associations between job demands, control, and strain and SA/DP were only observed in the group with > 365 days of SA/DP during the three follow-up years. This finding highlights the importance of distinguishing between short-term and long-term SA/DP, as the latter could indicate the path to more permanent labor market marginalization and appear to be influenced by different factors than short-term outcomes, such as, for instance, health status, disorder severity, and work ability [50]. Additionally, while some individuals with > 365 SA/DP days may experience continuous long-term absence, others may have recurrent shorter SA spells, alternating between return to work and starting a new SA spell. These transitions can, at least partially, reflect a detrimental workplace environment.

Regarding the unemployment associations, prior research has shown that having any mental disorder, including a CMD, is associated with an increased risk of not being employed in the future [51]. As for the role of occupational factors, a Swedish twin study found that higher levels of job control were associated with a reduced risk of subsequent unemployment in the general population [52]. However, to the best of our knowledge, our study is the first to investigate how job demands, control, and strain affect subsequent unemployment outcomes specifically among individuals with SA due to CMDs. As with SA/DP outcomes, we found that high-strain (high demands/low control), passive (low demands/low control), and low-control jobs were associated with medium and long-term unemployment in this population. Low control appears to be the common factor contributing to post-SA unemployment in this population, as was also suggested for the SA/DP outcomes. This may reflect a lack of flexibility over one’s working hours and pace, and limited opportunities to accommodate the needs of individuals on SA due to CMDs, hindering their return-to-work process and contributing to labor market marginalization [14]. It is also plausible that jobs characterized by low control are inherently more precarious, leading to reduced physical and mental health and increased unemployment [53].

Finally, regarding the associations between the structural occupational factors, namely occupational sector and branch, we found that individuals in the public sector were less likely to experience medium and long-term unemployment but more likely to have long-term SA/DP following an SA spell due to a CMD. While not directly aligned given the differences in study populations, our results are consistent with some previous findings showing that workers in the public sector are more likely to have a CMD [26] and are also more likely to experience SA due to CMDs compared to those in the private sector [28].

In Sweden, many companies, particularly within health and social care, are private companies contracted to deliver publicly financed health and social care. Therefore, we decided to also investigate how working in different branches might affect labor market outcomes after SA due to CMDs, as opposed to only examining the occupational sector. We found that working in the education and public administration branch, as well as in the health and social care branch, was associated with a lower likelihood of having both medium and long-term unemployment, yet a higher likelihood of experiencing medium and long-term SA/DP. The findings regarding the SA/DP outcomes are in line with several previous studies [28, 29] and may reflect higher workplace mental strain [28]. On the other hand, a lower likelihood of unemployment in the public sector, the education and public administration branch, and the health and social care branch is likely due to greater job security, more stable employment conditions, and a higher demand for skilled professionals. However, since employment in education and public administration and in health and social care was highly correlated with employment in the public sector, we were unable to determine whether the unemployment results were attributable to sector or branch-related factors. This warrants further assessment with a larger number of individuals employed in these branches within the private sector.

### Strengths and Limitations

First, this large-scale, population-based study included 79,673 working-age individuals and used high-quality national register data, resulting in a representative sample that enhances the generalizability of the findings to the broader population with SA benefits due to CMDs. Second, since assessing unemployment alone tends to undermine labor market marginalization in this population, including health-related marginalization using SA/DP measures contributes to a more comprehensive understanding of how individuals with SA due to CMDs can be excluded from the labor market. Third, we assessed the psychosocial work environment at the occupational level using the SweJEM, which minimizes the subjectivity of the self-reported individual measures, as the individual scores were averaged out for all individuals within a specific occupational group. Lastly, we categorized job demands and job control into low, medium, or high, which allowed us to better understand how more extreme conditions contribute to unemployment and SA/DP following an SA spell due to a CMD. If we had dichotomized job demands and job control scores into low and high, we could have diluted the effects, since most individuals in this study and several previous studies fell in the middle of the score scales [42].

Our study also has several limitations. First, we did not have information about SA spells lasting 14 days or less, as in Sweden, the benefits for these spells are generally paid by the employer. Although this lack of data did not affect the inclusion of individuals in our study population, it meant that shorter spells, likely resulting from lower symptom severity, were not part of the assessment of SA/DP days during the follow-up. Second, because data from primary care settings were unavailable, our analyses did not include mental and somatic comorbidities diagnosed in these settings. However, more severe mental and somatic disorders and neurodevelopmental conditions—which are likely to have the greatest impact on the association estimates—are generally diagnosed in specialized healthcare settings and, thus, were included in our study. Third, we also did not have data on several important characteristics, such as personality traits, prior job performance, and workplace accommodations and support, which could have affected the results. Fourth, the occupational-level assessment of job demands, control, and strain meant that individual variation could not be captured. As a result, all individuals within the same occupation had identical values for job demands, control, and strain, even though their specific work environments may have differed. While the occupational-level assessment allowed us to measure the core components of job demands, control, and strain that are specific to a particular occupation, it could have attenuated the associations. Finally, we were unable to assess potential changes in individuals’ occupations during the follow-up due to the lack of reliable data. However, such occupational-level changes are likely uncommon, as they often require additional training and education, and may take longer than our study’s follow-up.

The findings of this study are only generalizable to countries with social security systems similar to Sweden, as well as with comparable job markets. Additionally, while the generalizability may be limited across time periods due to the potential changes in the labor market, it is likely that the core components of job demands, control, and strain characteristic to each occupation remain relatively stable.

## Conclusion

Job demands, job control, and job strain, and structural occupational factors, such as occupational sector and branch, affect subsequent labor market outcomes following a sickness absence spell due to CMDs. Particularly, high-strain jobs, characterized by high demands and low control, and passive jobs, characterized by both low demands and low control, were found to be associated with medium and long-term unemployment and long-term sickness absence and disability pension, suggesting potential labor market marginalization. Future studies are necessary to further investigate these associations and inform effective interventions to promote long-term labor market inclusion for individuals with sickness absence due to CMDs.

## Supplementary Information

Below is the link to the electronic supplementary material.

Supplementary file1 (PDF 308 KB)

## Data Availability

The data used in this study cannot be made publicly available due to privacy regulations. According to the General Data Protection Regulation, the Swedish law SFS 2018:218, the Swedish Data Protection Act, the Swedish Ethical Review Act, and the Public Access to Information and Secrecy Act, these types of sensitive data can only be made available for specific purposes, including research, that meets the criteria for access to this type of sensitive and confidential data as determined by a legal review. Readers may contact Professor Ellenor Mittendorfer-Rutz (ellenor.mittendorfer-rutz@ki.se) regarding the data.
