# Supplementary material for: Occupational Factors and Labor Market Outcomes Among Individuals with Sickness Absence due to Common Mental Disorders: A Population-Wide Cohort Study
Source: J Occup Rehabil. 2025 Nov 27;36(4):1221–36. doi: 10.1007/s10926-025-10348-6 (PMC13759578; doi:10.1007/s10926-025-10348-6)

**Journal of Occupational Rehabilitation**

# Occupational Factors and Labor Market Outcomes Among Individuals with Sickness Absence due to Common Mental Disorders: A Population-Wide Cohort Study

Gerda Stutaite[1], Ellenor Mittendorfer-Rutz[1], Magnus Helgesson[1,2], Kristin Farrants[1], Katalin Gémes[1]

[1]Division of Insurance Medicine, Department of Clinical Neuroscience, Karolinska Institutet, SE-171 77 Stockholm, Sweden.

[2]Department of Public Health and Caring Sciences, Uppsala University, Box 564, 751 22, Uppsala, Sweden.

Corresponding author:

Gerda Stutaite

Division of Insurance Medicine

Department of Clinical Neuroscience

Karolinska Institutet

SE-171 77 Stockholm

Sweden

Email: gerda.stutaite@ki.se

Phone: +46 73-946 02 30

## Supplementary Information

### Data sources

We used the following national Swedish registers and databases, which are linked together by a unique identification number assigned to each individual:

- The "Micro-Data for Analyses of Social Insurance (MiDAS)" [1] for information about diagnoses, dates, and grades (part-time versus full-time) of sickness absence and disability pension benefits.
- The "Longitudinal Integration Database for Health Insurance and Labor Market Studies (LISA)" [2] for information about sociodemographic, socioeconomic, and occupational factors, including occupation, occupational sector, and branch.
- The National Patient Register [3] for information about primary and secondary diagnoses from specialized outpatient care visits and inpatient care stays to identify mental and somatic comorbidities.
- The Causes of Death Register [4] for information about dates of death.
- The Prescribed Drug Register [5] for information about dispensed psychotropic medications.

### References:


1. Österlund N. MiDAS - Sickness Benefit and Rehabilitation Allowance. Swedish Social Insurance Agency. 2011;

2. Ludvigsson JF, Svedberg P, Olén O, Bruze G, Neovius M. The longitudinal integrated database for health insurance and labour market studies (LISA) and its use in medical research. European Journal of Epidemiology [Internet]. 2019;34(4):423–37. Available from: 10.1007/s10654-019-00511-8

3. Ludvigsson JF, Andersson E, Ekbom A, Feychting M, Kim JL, Reuterwall C, et al. External review and validation of the Swedish national inpatient register. BMC Public Health [Internet]. 2011;11(1). Available from: 10.1186/1471-2458-11-450

4. Brooke HL, Talbäck M, Hörnblad J, Johansson LA, Ludvigsson JF, Druid H, et al. The Swedish cause of death register. European Journal of Epidemiology [Internet]. 2017;32(9):765–73. Available from: 10.1007/s10654-017-0316-1

5. Wettermark B, Hammar N, Fored CM, Leimanis A, Otterblad Olausson P, Bergman U, et al. The new Swedish Prescribed Drug Register--opportunities for

pharmacoepidemiological research and experience from the first six months. Pharmacoepidemiology and Drug Safety [Internet]. 2007;16(7):726–35. Available from: 10.1002/pds.1294

**Table 1. Baseline Characteristics of Individuals Included in the Unemployment (UE) Analysis (n = 78,900 (99.0%)) and Those Excluded due to Emigration or Death (n = 773 (1.0%)).**

| Characteristic | n Included % | n Excluded % |
|---|---|---|
| **Sex:** | | |
| Female | 56,488 (71.6) | 418 (54.1) |
| Male | 22,412 (28.4) | 355 (45.9) |
| **Age:** | | |
| 25-29 | 9,369 (11.9) | 112 (14.5) |
| 30-34 | 12,390 (15.7) | 121 (15.6) |
| 35-39 | 15,276 (19.4) | 108 (14.0) |
| 40-44 | 15,187 (19.2) | 132 (17.1) |
| 45-49 | 13,778 (17.5) | 158 (20.4) |
| 50-55 | 12,900 (16.3) | 142 (18.4) |
| **Country of birth:** | | |
| Sweden | 68,597 (86.9) | 528 (68.3) |
| Other Nordic | 1,696 (2.2) | 78 (10.1) |
| Other European | 4,020 (5.1) | 81 (10.5) |
| Rest of the World | 4,587 (5.8) | 86 (11.1) |
| **Education level:** | | |
| Elementary (< 10 years) | 6,591 (8.3) | 99 (12.8) |
| High school (10-12 years) | 36,266 (46.0) | 320 (41.4) |
| University or college (> 12 years) | 36,043 (45.7) | 354 (45.8) |
| **Family composition:** | | |
| Living without a partner and without children | 25,654 (32.5) | 357 (46.2) |
| Living without a partner and with children | 9,017 (11.4) | 69 (8.9) |
| Living with a partner and without children | 9,881 (12.5) | 101 (13.1) |
| Living with a partner and with children | 34,348 (43.6) | 246 (31.8) |
| **Type of living area:** | | |
| City | 31,183 (39.5) | 358 (46.3) |
| Town or suburban area | 33,187 (42.1) | 304 (39.3) |
| Rural area | 14,530 (18.4) | 111 (14.4) |
| **Occupational sector:** | | |
| Private | 45,469 (57.6) | 514 (66.5) |
| Public | 33,431 (42.4) | 259 (33.5) |
| **Occupational branch:** | | |
| Production, industrial, and resource-based | 10,838 (13.7) | 127 (16.4) |
| Trade, transport, and storage | 12,316 (15.6) | 122 (15.8) |
| Information, financial, and business services | 14,282 (18.1) | 177 (22.9) |
| Hospitality and personal services | 3,597 (4.6) | 42 (5.4) |
| Education and public administration | 17,820 (22.6) | 132 (17.1) |
| Health and social services | 20,047 (25.4) | 173 (22.4) |
| **Job demands (SweJEM):** | | |
| Low | 11,914 (15.1) | 145 (18.8) |
| Medium | 37,164 (47.1) | 314 (40.6) |
| High | 29,822 (37.8) | 314 (40.6) |

| | | |
|---|---|---|
| **Job control (SweJEM):** | | |
| Low | 26,379 (33.4) | 277 (35.8) |
| Medium | 30,352 (38.5) | 269 (34.8) |
| High | 22,169 (28.1) | 227 (29.4) |
| **Job strain (SweJEM):** | | |
| Active jobs (high demands/high control) | 12,165 (15.4) | 137 (17.7) |
| Intermediate strain | 57,041 (72.3) | 500 (64.7) |
| Passive jobs (low demands/low control) | 6,290 (8.0) | 81 (10.5) |
| High-strain jobs (high demands/low control) | 3,221 (4.1) | 52 (6.7) |
| **Any unemployment days:** | | |
| Yes | 5,924 (7.5) | 76 (9.8) |
| No | 72,976 (92.5) | 697 (90.2) |
| **SA history:** | | |
| 0 days (no SA history) | 67,101 (85.0) | 649 (84.0) |
| 0 days < SA history ≤ 30 days | 4,848 (6.1) | 30 (3.9) |
| 30 days < SA history ≤ 90 days | 4,120 (5.2) | 63 (8.2) |
| 90 days < SA history ≤ 180 days | 1,761 (2.2) | 20 (2.6) |
| 180 days < SA history | 1,070 (1.4) | 11 (1.4) |
| **Index SA diagnosis:** | | |
| Depressive disorders (F32-F33) | 29,476 (37.4) | 361 (46.7) |
| Anxiety disorders and OCD (F40-F41 and F42) | 9,828 (12.5) | 109 (14.1) |
| Stress-induced disorders (F43) | 39,596 (50.2) | 303 (39.2) |
| **Index SA grade:** | | |
| Full-time (100%) | 71,157 (90.2) | 713 (92.2) |
| Part-time (0%, 25%, or 75%) | 7,743 (9.8) | 60 (7.8) |
| **Net index SA days:** | | |
| 30 days < SA days ≤ 90 days | 40,419 (51.2) | 387 (50.1) |
| 90 days < SA days ≤ 180 days | 17,425 (22.1) | 176 (22.8) |
| 180 days < SA days | 21,056 (26.7) | 210 (27.2) |
| **Mental comorbidities:** | | |
| Yes | 5,524 (7.0) | 117 (15.1) |
| No | 73,376 (93.0) | 656 (84.9) |
| **Somatic comorbidities:** | | |
| Yes | 34,122 (43.2) | 316 (40.9) |
| No | 44,778 (56.8) | 457 (59.1) |
| **Medications:** | | |
| Antidepressants (ATC: N06A): | | |
| No dispensations | 61,071 (77.4) | 489 (63.3) |
| One dispensation | 9,804 (12.4) | 116 (15.0) |
| Two or more dispensations | 8,025 (10.2) | 168 (21.7) |
| Other psychotropic medications: | | |
| No dispensations | 62,688 (79.5) | 501 (64.8) |
| One or more dispensations | 16,212 (20.5) | 272 (35.2) |

a. Abbreviations (in alphabetical order): ADHD = Attention Deficit Hyperactivity Disorder, ATC = Anatomical Therapeutic Chemical, CMDs = Common Mental Disorders, OCD = Obsessive Compulsive Disorder, SA = Sickness Absence, SweJEM = The Swedish Job Exposure Matrix.

b. Measurement timepoints: Age, education level, family composition, type of living area, occupation, and occupational sector and branch were assessed on December 31 of the year before the index SA began. UE days were measured for the entire year preceding the start of the index SA spell. SA/DP history was assessed in the two-year period prior to the exclusion window, which began one year before the index SA. Mental and somatic comorbidities were assessed within two years of the start of the index SA spell, and medication use was assessed six months before and three months after the index SA spell started.
c. Missing information was classified as the lowest education level – "elementary education (<10 years)."
d. Low-strain jobs are not included due to small observation counts.
e. Intermediate job strain: low demands/medium control, medium demands/low control, medium demands/medium control, medium demands/high control, high demands/medium control.
f. All diagnostic codes refer to the 10th version of the International Classification of Diseases (ICD-10) codes.
g. Other psychotropic medications: anxiolytics (ATC: N05B), hypnotics and sedatives (ATC: N05C), psychostimulants, ADHD agents, and nootropics (ATC: N06B), psycholeptics and psychoanaleptics in combination (ATC: N06C).

**Table 2. Distribution of Exposure and Covariate Categories Across the Three Outcome Categories for Unemployment (UE) Days (n total = 78,900).**

| Total UE Days During Follow-Up | None (0) n = 60,673 | 0 < UE Days ≤ 180 n = 11,045 | > 180 UE Days n = 7,182 | Overall n = 78,900 |
|---|---|---|---|---|
| **Main exposures** | | | | |
| **Job strain (SweJEM):** | | | | |
| Intermediate | 44,301 (73.2%) | 7,956 (72.2%) | 4,784 (66.8%) | 57,041 (72.5%) |
| Active jobs (high demands/high control) | 9,824 (16.2%) | 1,394 (12.6%) | 947 (13.2%) | 12,165 (15.5%) |
| Passive jobs (low demands/low control) | 4,182 (6.9%) | 1,080 (9.8%) | 1,028 (14.4%) | 6,290 (8.0%) |
| High-strain jobs (high demands/low control) | 2,228 (3.7%) | 591 (5.4%) | 402 (5.6%) | 3,221 (4.1%) |
| **Job demands (SweJEM):** | | | | |
| Low | 8,356 (13.8%) | 1,947 (17.6%) | 1,611 (22.4%) | 11,914 (15.1%) |
| Medium | 28,073 (46.3%) | 5,607 (50.8%) | 3,484 (48.5%) | 37,164 (47.1%) |
| High | 24,244 (40.0%) | 3,491 (31.6%) | 2,087 (29.1%) | 29,822 (37.8%) |
| **Job control (SweJEM):** | | | | |
| Low | 18,784 (31.0%) | 4,445 (40.2%) | 3,150 (43.9%) | 26,379 (33.4%) |
| Medium | 24,050 (39.6%) | 4,044 (36.6%) | 2,258 (31.4%) | 30,352 (38.5%) |
| High | 17,839 (29.4%) | 2,556 (23.1%) | 1,774 (24.7%) | 22,169 (28.1%) |
| **Occupational sector:** | | | | |
| Private | 31,872 (52.5%) | 7,927 (71.8%) | 5,670 (78.9%) | 45,469 (57.6%) |
| Public | 28,801 (47.5%) | 3,118 (28.2%) | 1,512 (21.1%) | 33,431 (42.4%) |
| **Occupational branch:** | | | | |
| Production, industrial, and resource-based | 7,923 (13.1%) | 1,642 (14.9%) | 1,273 (17.7%) | 10,838 (13.7%) |
| Trade, transport, and storage | 8,474 (14.0%) | 2,249 (20.4%) | 1,593 (22.2%) | 12,316 (15.6%) |
| Information, financial, and business services | 1,0191 (16.8%) | 2,322 (21.0%) | 1,769 (24.6%) | 14,282 (18.1%) |
| Hospitality and personal services | 2,370 (3.9%) | 721 (6.5%) | 506 (7.0%) | 3,597 (4.6%) |
| Education and public administration | 15,088 (24.9%) | 1,842 (16.7%) | 890 (12.4%) | 17,820 (22.6%) |
| Health and social services | 16,627 (27.4%) | 2,269 (20.5%) | 1,151 (16.0%) | 20,047 (25.4%) |
| **Other covariates** | | | | |
| **Sex:** | | | | |
| Female | 44,671 (73.6%) | 7,551 (68.4%) | 4,266 (59.4%) | 56,488 (71.6%) |
| Male | 16,002 (26.4%) | 3,494 (31.6%) | 2,916 (40.6%) | 22,412 (28.4%) |
| **Age:** | | | | |
| 25-29 | 6,655 (11.0%) | 1,713 (15.5%) | 1,001 (13.9%) | 9,369 (11.9%) |
| 30-34 | 9,388 (15.5%) | 1,826 (16.5%) | 1,176 (16.4%) | 12,390 (15.7%) |
| 35-39 | 11,842 (19.5%) | 2,109 (19.1%) | 1,325 (18.4%) | 15,276 (19.4%) |
| 40-44 | 11,847 (19.5%) | 2,024 (18.3%) | 1,316 (18.3%) | 15,187 (19.2%) |
| 45-49 | 10,690 (17.6%) | 1,797 (16.3%) | 1,291 (18.0%) | 13,778 (17.5%) |
| 50-55 | 10,251 (16.9%) | 1,576 (14.3%) | 1,073 (14.9%) | 12,900 (16.3%) |
| **Country of birth:** | | | | |
| Sweden | 53,438 (88.1%) | 9,415 (85.2%) | 5,744 (80.0%) | 68,597 (86.9%) |
| Outside Sweden | 7,235 (11.9%) | 1,630 (14.8%) | 1,438 (20.0%) | 10,303 (13.1%) |
| **Education level:** | | | | |
| Elementary (<10 years) | 4,394 (7.2%) | 1,243 (11.3%) | 954 (13.3%) | 6,591 (8.4%) |
| High school (10-12 years) | 26,822 (44.2%) | 5,609 (50.8%) | 3,835 (53.4%) | 36,266 (46.0%) |
| University or college (>12 years) | 29,457 (48.6%) | 4,193 (38.0%) | 2,393 (33.3%) | 36,043 (45.7%) |
| **Type of living area:** | | | | |
| City | 23,659 (39.0%) | 4,408 (39.9%) | 3,116 (43.4%) | 31,183 (39.5%) |
| Suburban or town | 25,842 (42.6%) | 4,527 (41.0%) | 2,818 (39.2%) | 33,187 (42.1%) |
| Rural | 11,172 (18.4%) | 2,110 (19.1%) | 1,248 (17.4%) | 14,530 (18.4%) |
| **Mental comorbidities:** | | | | |
| None | 57,052 (94.0%) | 9,897 (89.6%) | 6,427 (89.5%) | 73,376 (93.0%) |
| Other CMDs and burnout | 2,471 (4.1%) | 711 (6.4%) | 477 (6.6%) | 3,659 (4.6%) |
| Other mental comorbidities | 1,150 (1.9%) | 437 (4.0%) | 278 (3.9%) | 1,865 (2.4%) |
| **Somatic comorbidities:** | | | | |
| None | 34,520 (56.9%) | 6,223 (56.3%) | 4,035 (56.2%) | 44,778 (56.8%) |
| One or more | 26,153 (43.1%) | 4,822 (43.7%) | 3,147 (43.8%) | 34,122 (43.2%) |
| **SA history:** | | | | |
| No | 51,886 (85.5%) | 9,158 (82.9%) | 6,057 (84.3%) | 67,101 (85.0%) |
| Yes | 8,787 (14.5%) | 1,887 (17.1%) | 1,125 (15.7%) | 11,799 (15.0%) |
| **DP history:** | | | | |
| No | 60,434 (99.6%) | 10,957 (99.2%) | 7,144 (99.5%) | 78,535 (99.5%) |
| Yes | 239 (0.4%) | 88 (0.8%) | 38 (0.5%) | 365 (0.5%) |
| **Index SA diagnosis:** | | | | |
| Depressive disorders (F32-F33) | 21,770 (35.9%) | 4,651 (42.1%) | 3,055 (42.5%) | 29,476 (37.4%) |
| Anxiety disorders and OCD (F40-F41 and F42) | 7,228 (11.9%) | 1,564 (14.2%) | 1,036 (14.4%) | 9,828 (12.5%) |
| Stress-induced disorders (F43) | 31,675 (52.2%) | 4,830 (43.7%) | 3,091 (43.0%) | 39,596 (50.2%) |

a. Abbreviations (in alphabetical order): CMDs = Common Mental Disorders, DP = Disability Pension, OCD = Obsessive Compulsive Disorder, SA = Sickness Absence, SweJEM = The Swedish Job Exposure Matrix.
b. Measurement timepoints: Age, education level, type of living area, and occupational sector and branch were assessed on December 31 of the year before the index SA began. SA/DP history was assessed in the two-year period prior to the exclusion window, which began one year before the index SA. Mental and somatic comorbidities were assessed within two years of the start of the index SA spell.
c. Intermediate job strain: low demands/medium control, medium demands/low control, medium demands/medium control, medium demands/high control, high demands/medium control.
d. Production, industrial, and resource-based branch: agriculture, forestry, fishing, manufacturing, extraction, energy supply, environmental services, and construction.
e. All diagnostic codes refer to the 10th version of the International Classification of Diseases (ICD-10) codes.

**Table 3. Distribution of Exposure and Covariate Categories Across the Four Outcome Categories for Sickness Absence (SA) and Disability Pension (DP) Days (n total = 78,967).**

| **Total SA/DP Days During Follow-Up** | **30 < Days ≤ 90**<br>**n = 26,635** | **90 < Days ≤ 180**<br>**n = 19,335** | **180 < Days ≤ 365**<br>**n = 16,388** | **> 365 Days**<br>**n = 16,609** | **Overall**<br>**n = 78,967** |
|---|---|---|---|---|---|
| **Main exposures** | | | | | |
| **Job strain:** | | | | | |
| Intermediate | 19,016 (71.6%) | 13,994 (72.5%) | 11,941 (73.1%) | 12,134 (73.2%) | 57,085 (72.5%) |
| Active jobs (high demands/high control) | 4,373 (16.5%) | 3,090 (16.0%) | 2,610 (16.0%) | 2,100 (12.7%) | 12,173 (15.5%) |
| Passive jobs (low demands/low control) | 2,124 (8.0%) | 1,434 (7.4%) | 1,170 (7.2%) | 1,572 (9.5%) | 6,300 (8.0%) |
| High-strain jobs (high demands/low control) | 1,059 (4.0%) | 774 (4.0%) | 623 (3.8%) | 769 (4.6%) | 3,225 (4.1%) |
| **Job demands:** | | | | | |
| Low | 3,984 (15.0%) | 2,799 (14.5%) | 2,294 (14.0%) | 2,860 (17.2%) | 11,937 (15.1%) |
| Medium | 12,402 (46.6%) | 9,022 (46.7%) | 7,699 (47.0%) | 8,068 (48.6%) | 37,191 (47.1%) |
| High | 10,249 (38.5%) | 7,514 (38.9%) | 6,395 (39.0%) | 5,681 (34.2%) | 29,839 (37.8%) |
| **Job control:** | | | | | |
| Low | 8,466 (31.8%) | 6,167 (31.9%) | 5,265 (32.1%) | 6,513 (39.2%) | 26,411 (33.4%) |
| Medium | 10,113 (38.0%) | 7,509 (38.8%) | 6,441 (39.3%) | 6,312 (38.0%) | 30,375 (38.5%) |
| High | 8,056 (30.2%) | 5,659 (29.3%) | 4,682 (28.6%) | 3,784 (22.8%) | 22,181 (28.1%) |
| **Occupational sector:** | | | | | |
| Private | 15,913 (59.7%) | 11,147 (57.7%) | 8,985 (54.8%) | 9,465 (57.0%) | 45,510 (57.6%) |
| Public | 10,722 (40.3%) | 8,188 (42.3%) | 7,403 (45.2%) | 7,144 (43.0%) | 33,457 (42.4%) |
| **Occupational branch:** | | | | | |
| Production, industrial, and resource-based | 3,928 (14.7%) | 2,751 (14.2%) | 2,108 (12.9%) | 2,069 (12.5%) | 10,856 (13.7%) |
| Trade, transport, and storage | 4,236 (15.9%) | 3,017 (15.6%) | 2,424 (14.8%) | 2,645 (15.9%) | 12,322 (15.6%) |
| Information, financial, and business services | 5,091 (19.1%) | 3,448 (17.8%) | 2,885 (17.6%) | 2,867 (17.3%) | 14,291 (18.1%) |
| Hospitality and personal services | 1,193 (4.5%) | 817 (4.2%) | 679 (4.1%) | 909 (5.5%) | 3,598 (4.6%) |
| Education and public administration | 5,855 (22.0%) | 4,503 (23.3%) | 3,899 (23.8%) | 3,573 (21.5%) | 17,830 (22.6%) |
| Health and social services | 6,332 (23.8%) | 4,799 (24.8%) | 4,393 (26.8%) | 4,546 (27.4%) | 20,070 (25.4%) |
| **Other covariates** | | | | | |
| **Sex:** | | | | | |
| Female | 18,175 (68.2%) | 13,846 (71.6%) | 12,283 (75.0%) | 12,217 (73.6%) | 56,521 (71.6%) |
| Male | 8,460 (31.8%) | 5,489 (28.4%) | 4,105 (25.0%) | 4,392 (26.4%) | 22,446 (28.4%) |
| **Age:** | | | | | |
| 25-29 | 3,442 (12.9%) | 2,436 (12.6%) | 1,804 (11.0%) | 1,692 (10.2%) | 9,374 (11.9%) |
| 30-34 | 4,344 (16.3%) | 3,127 (16.2%) | 2,533 (15.5%) | 2,392 (14.4%) | 12,396 (15.7%) |
| 35-39 | 5,226 (19.6%) | 3,782 (19.6%) | 3,148 (19.2%) | 3,127 (18.8%) | 15,283 (19.4%) |
| 40-44 | 4,969 (18.7%) | 3,706 (19.2%) | 3,245 (19.8%) | 3,275 (19.7%) | 15,195 (19.2%) |
| 45-49 | 4,498 (16.9%) | 3,239 (16.8%) | 2,906 (17.7%) | 3,158 (19.0%) | 13,801 (17.5%) |
| 50-55 | 4,156 (15.6%) | 3,045 (15.7%) | 2,752 (16.8%) | 2,965 (17.9%) | 12,918 (16.4%) |
| **Country of birth:** | | | | | |
| Sweden | 23,231 (87.2%) | 16,979 (87.8%) | 14,325 (87.4%) | 14,116 (85.0%) | 68,651 (86.9%) |
| Outside Sweden | 3,404 (12.8%) | 2,356 (12.2%) | 2,063 (12.6%) | 2,493 (15.0%) | 10,316 (13.1%) |
| **Education level:** | | | | | |
| Elementary (<10 years) | 2,066 (7.8%) | 1,442 (7.5%) | 1,267 (7.7%) | 1,828 (11.0%) | 6,603 (8.4%) |
| High school (10-12 years) | 11,945 (44.8%) | 8,732 (45.2%) | 7,475 (45.6%) | 8,150 (49.1%) | 36,302 (46.0%) |
| University or college (>12 years) | 12,624 (47.4%) | 9,161 (47.4%) | 7,646 (46.7%) | 6,631 (39.9%) | 36,062 (45.7%) |
| **Type of living area:** | | | | | |
| City | 10,910 (41.0%) | 7,817 (40.4%) | 6,294 (38.4%) | 6,186 (37.2%) | 31,207 (39.5%) |
| Suburban or town | 11,052 (41.5%) | 7,929 (41.0%) | 7,048 (43.0%) | 7,185 (43.3%) | 33,214 (42.1%) |
| Rural | 4,673 (17.5%) | 3,589 (18.6%) | 3,046 (18.6%) | 3,238 (19.5%) | 14,546 (18.4%) |
| **Mental comorbidities:** | | | | | |
| None | 25,254 (94.8%) | 18,219 (94.2%) | 15,224 (92.9%) | 14,728 (88.7%) | 73,425 (93.0%) |
| Other CMDs and burnout | 934 (3.5%) | 748 (3.9%) | 762 (4.6%) | 1,222 (7.4%) | 3,666 (4.6%) |
| Other mental comorbidities | 447 (1.7%) | 368 (1.9%) | 402 (2.5%) | 659 (4.0%) | 1,876 (2.4%) |
| **Somatic comorbidities:** | | | | | |
| None | 16,129 (60.6%) | 11,052 (57.2%) | 8,869 (54.1%) | 8,767 (52.8%) | 44,817 (56.8%) |
| One or more | 10,506 (39.4%) | 8,283 (42.8%) | 7,519 (45.9%) | 7,842 (47.2%) | 34,150 (43.2%) |
| **SA history:** | | | | | |
| No | 23,745 (89.1%) | 16,626 (86.0%) | 13,640 (83.2%) | 13,139 (79.1%) | 67,150 (85.0%) |
| Yes | 2,890 (10.9%) | 2,709 (14.0%) | 2,748 (16.8%) | 3,470 (20.9%) | 11,817 (15.0%) |
| **DP history:** | | | | | |
| No | 26,574 (99.8%) | 19,286 (99.7%) | 16,317 (99.6%) | 16,425 (98.9%) | 78,602 (99.5%) |
| Yes | 61 (0.2%) | 49 (0.3%) | 71 (0.4%) | 184 (1.1%) | 365 (0.5%) |
| **Index SA diagnosis:** | | | | | |
| Depressive disorders (F32-F33) | 8,668 (32.5%) | 7,253 (37.5%) | 6,323 (38.6%) | 7,270 (43.8%) | 29,514 (37.4%) |
| Anxiety disorders and OCD (F40-F41 and F42) | 3,250 (12.2%) | 2,296 (11.9%) | 2,001 (12.2%) | 2,288 (13.8%) | 9,835 (12.5%) |
| Stress-induced disorders (F43) | 14,717 (55.3%) | 9,786 (50.6%) | 8,064 (49.2%) | 7,051 (42.5%) | 39,618 (50.2%) |

a. Abbreviations (in alphabetical order): CMDs = Common Mental Disorders, DP = Disability Pension, OCD = Obsessive Compulsive Disorder, SA = Sickness Absence, SweJEM = The Swedish Job Exposure Matrix.

b. Measurement timepoints: Age, education level, type of living area, and occupational sector and branch were assessed on December 31 of the year before the index SA began. The SA and DP history was assessed within two years of the index SA start, that is, one year before the exclusion period started. Mental and somatic comorbidities were assessed within two years of the start of the index SA spell.

c. Intermediate job strain: low demands/medium control, medium demands/low control, medium demands/medium control, medium demands/high control, high demands/medium control.

d. Production, industrial, and resource-based branch: agriculture, forestry, fishing, manufacturing, extraction, energy supply, environmental services, and construction.

e. All diagnostic codes refer to the 10th version of the International Classification of Diseases (ICD-10) codes.

**Table 4. Multinomial Logistic Regression Results for Job Strain and Unemployment (UE) Days with the Individuals with Low-Strain Jobs (n = 183 (0.23%)) (total n = 78,900).** Model 1 was adjusted for sex and age, Model 2 was additionally adjusted for education, country of birth, and type of living area, and Model 3 was further adjusted for index SA diagnosis, SA/DP history, and mental and somatic comorbidities. Reference category: 0 UE days during the follow-up period.

| Occupational Characteristic | Outcome Category | Crude OR (95% CI) | Model 1 OR (95% CI) | Model 2 OR (95% CI) | Model 3 OR (95% CI) |
|---|---|---|---|---|---|
| **Job strain:** | | | | | |
| Intermediate | 0 < UE Days ≤ 180 | 1.00 (ref) | 1.00 (ref) | 1.00 (ref) | 1.00 (ref) |
| | > 180 UE Days | 1.00 (ref) | 1.00 (ref) | 1.00 (ref) | 1.00 (ref) |
| Low-strain jobs (low demands/high control) | 0 < UE Days ≤ 180 | 0.97 (0.63-1.49) | 0.93 (0.60-1.44) | 0.98 (0.63-1.52) | 0.98 (0.63-1.51) |
| | > 180 UE Days | 1.41 (0.89-2.23) | 1.27 (0.80-2.02) | 1.41 (0.89-2.24) | 1.42 (0.89-2.26) |
| Active jobs (high demands/high control) | 0 < UE Days ≤ 180 | 0.79 (0.74-0.84) | 0.79 (0.74-0.84) | 0.88 (0.83-0.94) | 0.91 (0.85-0.97) |
| | > 180 UE Days | 0.89 (0.83-0.96) | 0.84 (0.78-0.90) | 0.98 (0.91-1.06) | 1.01 (0.93-1.09) |
| Passive jobs (low demands/low control) | 0 < UE Days ≤ 180 | 1.44 (1.34-1.54) | 1.31 (1.22-1.41) | 1.14 (1.06-1.23) | 1.11 (1.03-1.20) |
| | > 180 UE Days | 2.28 (2.11-2.45) | 1.83 (1.70-1.98) | 1.50 (1.39-1.62) | 1.48 (1.36-1.60) |
| High-strain jobs (high demands/low control) | 0 < UE Days ≤ 180 | 1.48 (1.34-1.62) | 1.37 (1.25-1.51) | 1.28 (1.17-1.41) | 1.27 (1.15-1.39) |
| | > 180 UE Days | 1.67 (1.50-1.87) | 1.38 (1.23-1.54) | 1.22 (1.09-1.37) | 1.21 (1.08-1.36) |

a. Intermediate job strain: low demands/medium control, medium demands/low control, medium demands/medium control, medium demands/high control, high demands/medium control.

**Table 5. Multinomial Logistic Regression Results for Job Strain and Sickness Absence and Disability Pension (SA/DP) Days with the Individuals with Low-Strain Jobs (n = 184 (0.24%) (n total = 78,967).** Model 1 was adjusted for sex and age, Model 2 was additionally adjusted for education, country of birth, and type of living area, and Model 3 was further adjusted for index SA diagnosis, SA/DP history, and mental and somatic comorbidities. Reference category: 30 < SA/DP Days ≤ 90 during the follow-up period.

| Occupational Characteristic | Outcome Category | Crude OR (95% CI) | Model 1 OR (95% CI) | Model 2 OR (95% CI) | Model 3 OR (95% CI) |
|---|---|---|---|---|---|
| **Job strain:** | | | | | |
| Intermediate | 90 < SA/DP Days ≤ 180 | 1.00 (ref) | 1.00 (ref) | 1.00 (ref) | 1.00 (ref) |
| | 180 < SA/DP Days ≤ 365 | 1.00 (ref) | 1.00 (ref) | 1.00 (ref) | 1.00 (ref) |
| | > 365 SA/DP Days | 1.00 (ref) | 1.00 (ref) | 1.00 (ref) | 1.00 (ref) |
| Low-strain jobs (low demands/high control) | 90 < SA/DP Days ≤ 180 | 0.93 (0.63-1.37) | 0.95 (0.65-1.41) | 0.96 (0.65-1.41) | 0.97 (0.66-1.44) |
| | 180 < SA/DP Days ≤ 365 | 1.11 (0.76-1.64) | 1.18 (0.80-1.74) | 1.19 (0.81-1.76) | 1.22 (0.82-1.79) |
| | > 365 SA/DP Days | 0.85 (0.56-1.28) | 0.89 (0.59-1.36) | 0.93 (0.61-1.41) | 0.93 (0.61-1.43) |
| Active jobs (high demands/high control) | 90 < SA/DP Days ≤ 180 | 0.96 (0.91-1.01) | 0.98 (0.93-1.03) | 0.98 (0.93-1.04) | 1.00 (0.95-1.06) |
| | 180 < SA/DP Days ≤ 365 | 0.95 (0.90-1.00) | 0.98 (0.93-1.03) | 1.01 (0.95-1.06) | 1.04 (0.98-1.10) |
| | > 365 SA/DP Days | 0.75 (0.71-0.80) | 0.76 (0.72-0.81) | 0.85 (0.80-0.90) | 0.90 (0.85-0.96) |
| Passive jobs (low demands/low control) | 90 < SA/DP Days ≤ 180 | 0.92 (0.86-0.98) | 0.98 (0.91-1.05) | 0.98 (0.91-1.05) | 0.96 (0.89-1.04) |
| | 180 < SA/DP Days ≤ 365 | 0.88 (0.81-0.95) | 1.00 (0.93-1.08) | 0.98 (0.90-1.05) | 0.95 (0.88-1.03) |
| | > 365 SA/DP Days | 1.16 (1.08-1.24) | 1.31 (1.22-1.41) | 1.14 (1.06-1.23) | 1.09 (1.01-1.17) |
| High-strain jobs (high demands/low control) | 90 < SA/DP Days ≤ 180 | 0.99 (0.90-1.09) | 1.05 (0.95-1.16) | 1.05 (0.95-1.16) | 1.04 (0.94-1.15) |
| | 180 < SA/DP Days ≤ 365 | 0.94 (0.85-1.04) | 1.05 (0.95-1.17) | 1.04 (0.94-1.16) | 1.02 (0.92-1.13) |
| | > 365 SA/DP Days | 1.14 (1.03-1.25) | 1.26 (1.15-1.39) | 1.20 (1.08-1.32) | 1.15 (1.04-1.27) |

a. Intermediate job strain: low demands/medium control, medium demands/low control, medium demands/medium control, medium demands/high control, high demands/medium control.

**Fig 1 Study Timeline.** Visual representation for a hypothetical individual with an index sickness absence (SA) spell starting in 2012. Individuals with index SA spells in 2011 or 2013 would follow the same timeline, shifted one year earlier or later

Abbreviations (in alphabetical order): DP = Disability Pension, SA = Sickness Absence

Severe psychiatric or neurological disorders refer to specialized outpatient or inpatient care for psychosis spectrum disorders (ICD-10: F20-29), bipolar disorder (ICD-10: F30-F31), early-onset neurodegenerative disorders (ICD-10: F00-F03, G30-G32), anorexia nervosa (ICD-10: F50.0), and intellectual disabilities (ICD-10: F70-F79)

Mental and somatic comorbidities and psychotropic medications are listed in Table 1 in the main manuscript

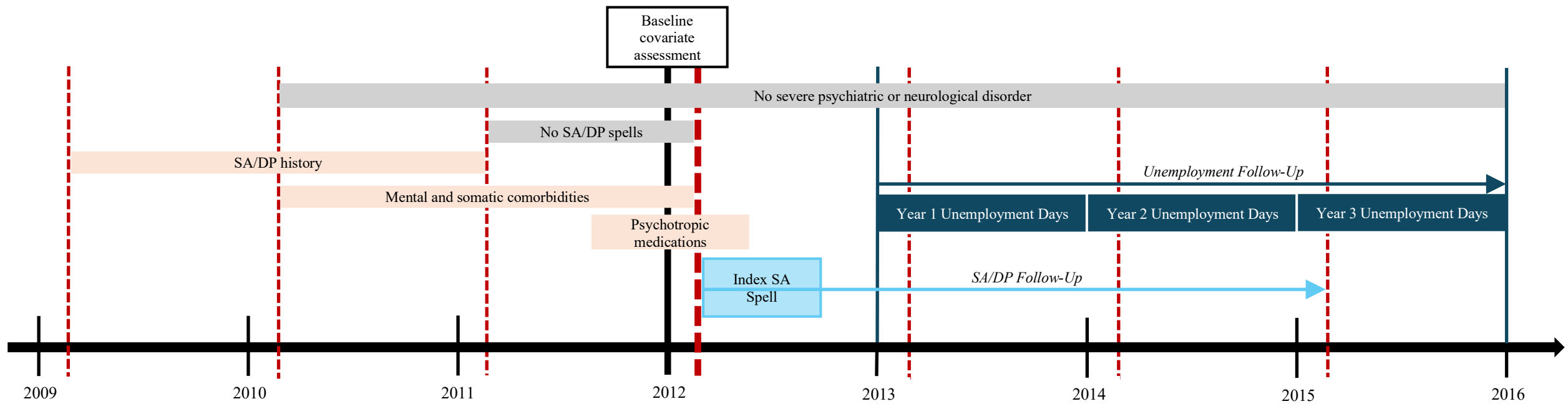

Supplement: Supplementary file 1 [file 10926_2025_10348_MOESM1_ESM.pdf]
